# National trends in adult cardiac surgery inpatient costs: A decade in review

**DOI:** 10.1016/j.xjon.2025.101551

**Published:** 2025-12-06

**Authors:** Troy N. Coaston, Amulya Vadlakonda, Kevin Tabibian, Esteban Aguayo, Sara Sakowitz, Saad Mallick, Richard J. Shemin, Peyman Benharash

**Affiliations:** a Department of Surgery, Cardiovascular Outcomes Research Laboratories (CORELAB), David Geffen School of Medicine at University of California, Los Angeles, Calif; b Division of Cardiac Surgery, Department of Surgery, David Geffen School of Medicine at University of California, Los Angeles, Calif

**Keywords:** cardiac surgery, inpatient costs, transcatheter valve procedures, health disparities, cost trends, resource use, health policy

## Abstract

**Objective:**

To characterize national trends in inpatient costs associated with adult cardiac surgery from 2013 to 2022 and identify factors independently associated with increased hospitalization expenditures.

**Methods:**

All hospitalizations entailing major cardiac operations (coronary artery bypass grafting, valve procedures [open and transcatheter], aortic repair) were identified in the 2013-2022 National Inpatient Sample. Temporal trends were evaluated using the Cuzick test for trend (nptrend), and multivariable linear regression was used to identify factors associated contemporary costs (2022).

**Results:**

Among an estimated 3,323,645 admissions, annual volume increased from 293,645 to 361,355 (nptrend = 0.01). Elective hospitalization costs increased from $8.1 to $12.9 billion (nptrend < 0.001), with median per-admission costs increasing from $41,000 to $48,000 (nptrend < 0.001). For nonelective hospitalizations, total costs rose from $8.3 to $10.4 billion (nptrend = 0.01), and median per-admission costs from $51,000 to $63,000 (nptrend < 0.001). The proportion of transcatheter valve procedures rose from 6.4% to 38.9% (nptrend < 0.001), whereas their median per-admission costs decreased. In 2022, Black race (β $9,300, 95% confidence interval [CI], $6700-$11,800) and care in the Western United States (β $12,800, 95% CI, $7600-$17,900) were associated with increased costs. Elective admission (β –$26,000, 95% CI, –$27,900 to –$24,200), older age (β –$1400 per decade, 95% CI, –$2100 to –$800), and lowest income quartile (β –$3200, 95% CI, –$5700 to –$800; ref: highest) were associated with lower costs.

**Conclusions:**

Inpatient cardiac surgery costs increased significantly over the decade. These trends, alongside increased use of transcatheter techniques and persistent disparities, underscore the need for systemic reform to ensure sustainable and equitable care.

**Figure:**
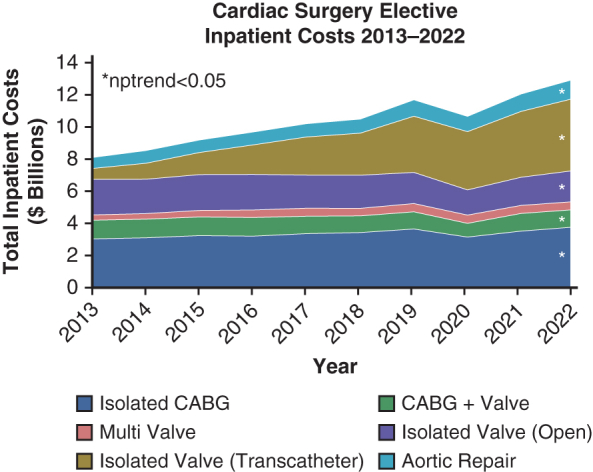
Cardiac surgery elective inpatient costs 2013-2022.

**Central Message**

From 2013 to 2022, inpatient cardiac surgery costs rose significantly. Transcatheter valve use grew 6-fold, whereas racial, regional, and economic disparities persisted, highlighting the need for reform.

**Perspective**

This study highlights increasing inpatient costs for cardiac surgery and identifies key drivers of variation, including procedure type, race, and region. The findings emphasize the need for innovation and policy reform to ensure cost-effective, equitable care and may inform strategies to align cardiac surgical spending with value-based care goals.

Health care expenditures in the United States continue to increase, accounting for nearly 17% of the gross domestic product in 2022, more than any other nation.^1^ In particular, total costs for all cardiovascular conditions are expected to increase from $627 billion in 2020 to $1.851 trillion in 2050.^2^

Cardiac surgery is a notably resource-intensive field, driven by procedural complexity, intensity of perioperative management, and potential for prolonged hospitalization.^3^ Despite the significant evolution of cardiac surgery over the past several decades, expenditures attributable to such operations at the large scale remain uncharacterized. The introduction of minimally invasive techniques including robotic and transcatheter technologies carry the promise of shorter hospitalizations, fewer complications, and ultimately cost reduction. However, older patients with more advanced comorbidities are increasingly considered surgical candidates, and care for patients with such complexity requires appropriate financial investment.^4^ Understanding the evolution of inpatient costs and identifying expenditure patterns is essential for optimizing resource allocation and informing policy development amid burgeoning health care costs.^5^

The present study sought to investigate national trends in inpatient cardiac surgical expenditures from over the past decade. We hypothesized that both episodic and overall hospitalization costs would have significantly increased over the past decade. Furthermore, we postulated that transcatheter valve operations would comprise a greater proportion of overall expenditures while decreasing in median per-admission costs.

## Methods

**Figure E1:**
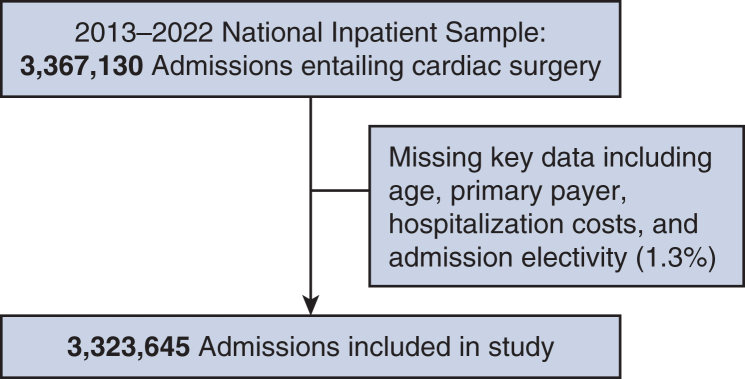
Identification of transcatheter cardiac valve procedures included in the study. Records lacking key variables—age, primary payer, hospitalization costs, or admission electivity—were excluded from the analysis.

This was a retrospective cohort study of the 2013-2022 National Inpatient Sample (NIS). As part of the Healthcare Cost and Utilization Project,^6^ the NIS is the largest all-payer database in the United States and provides accurate estimates for ∼97% of hospitalizations through survey weighting methodology. All admissions entailing major cardiac surgery (coronary artery bypass grafting [CABG], cardiac valve repair/replacement, and aortic repair), were identified in the NIS using previously validated *International Classification of Diseases, Ninth and Tenth Revisions,* codes.^7^^,^^8^ Transcatheter cardiac valve operations were similarly identified and included in the study.^7^ Records missing key data including age, primary payer, hospitalization costs, and admission electivity were excluded from analysis (Figure E1).

Patient (age, sex, race, admission electivity, and primary payer) and facility characteristics are reported as described in the NIS data dictionary.^6^ Subjects were stratified by age, primary payer, admission electivity, and operation type (CABG, isolated open cardiac valve procedures, isolated transcatheter cardiac valve procedures, concomitant CABG/valve operations, multiple valve operations, and aortic repair). The burden of chronic conditions was quantified using the Van Walraven modification of the Elixhauser Comorbidity Index.^9^ Inpatient costs were tabulated by applying center-specific cost-to-charge ratios and adjusted for inflation to the 2022 Personal Health Index.^10^ To generate national estimates, each admission's cost was multiplied by the corresponding discharge weight (ie, DISCWT) provided in the NIS, allowing for weighted aggregation across all study years.

The primary aim was to assess temporal trends in hospitalization costs. We also examined trends in acute mortality and factors independently associated with increased hospitalization costs.

Continuous and categorical variables are reported as medians with interquartile range or proportions, respectively. The Mann-Whitney *U* and Pearson χ^2^ tests were used to evaluate between-group differences. The Cuzick nonparametric test was used to determine the significance of temporal trends (nonparametric trend, or nptrend). Multivariable linear regression models were developed to identify clinically relevant factors independently associated with hospitalization costs (2022). Regression outputs are reported as β coefficients alongside 95% confidence intervals (CIs).

All analyses were completed using Stata 18.0 (StataCorp). Because of its deidentified nature, this study was deemed exempt from full review by the institutional review board at the University of California, Los Angeles (no. 17-001112, July 26, 2017). The institutional review board waived written patient consent for inclusion in the study. Statistical significance was set at α = 0.05.

## Results

Of an estimated 3,323,645 admissions entailing cardiac surgery from 2013 to 2022, 59.9% were elective. The overall annual volume of hospitalizations increased from 293,645 in 2013 to 361,355 in 2022 (nptrend = 0.01). In addition, there was an increase in the proportion of elective hospitalizations from 55.8% to 64.6% (nptrend < 0.001). Median age (67 [59-75] to 70 years [62-77]), comorbidity burden (Elixhauser: 4 [3-5] to 5 [3-6]), and the percentage of patients of White race (74.5% to 77.3%; all nptrend < 0.001; Table 1) increased over the study duration.

**Table 1:** Trends in demographic and hospital characteristics of patients undergoing cardiac surgery from 2013 to 2022

Parameter	Overall (N = 3,323,645)	2013 (n = 293,645)	2022 (n = 361,355)	nptrend
Age, y, median [IQR]	68 [60-76]	67 [59-75]	70 [62-77]	<0.001
Female, %	32.2	32.0	32.5	0.03
Elixhauser, median [IQR]	4 [3-6]	4 [3-5]	5 [3-6]	<0.001
Length of stay, d, median [IQR]	7 [5-11]	8 [6-11]	6 [3-10]	<0.001
Race, %				
White	76.4	74.5	77.3	<0.001
Black	6.7	6.7	6.6	0.89
Hispanic	6.7	6.2	7.2	<0.001
Asian	2.6	2.2	3.1	<0.001
Other	7.5	10.5	5.6	<0.001
Income quartile, %				
76th-100th	22.5	21.8	23.3	<0.001
51st-75th	25.3	25.3	25.6	<0.001
26th-50th	26.7	26.8	26.3	0.01
0-25th	25.5	26.1	24.8	<0.001
Insurance coverage, %				
Private	27.5	29.3	26.0	<0.001
Medicare	60.2	57.7	62.3	<0.001
Medicaid	7.0	5.8	6.8	0.14
Other	5.3	7.2	4.9	<0.001
Operation type, %				
Isolated CABG	48.8	55.3	44.3	<0.001
CABG + valve	7.9	10.4	11.9	<0.001
Multi valve	2.7	2.3	2.4	0.03
Isolated valve (open)	15.5	20.2	11.9	<0.001
Isolated valve (transcatheter)	18.4	4.6	29.7	<0.001
Aortic repair	6.6	7.1	6.7	<0.001
Elective admission, %	59.9	55.8	64.6	<0.001
Hospital bed size, %				
Large	67.1	73.9	64.5	<0.001
Medium	23.3	19.5	24.4	<0.001
Small	9.6	6.7	11.1	<0.001
Teaching status, %				
Nonmetropolitan	2.3	3.2	2.1	<0.001
Nonteaching metropolitan	14.5	29.2	8.4	<0.001
Teaching metropolitan	83.3	67.6	89.5	<0.001

Reported as proportions unless otherwise noted. *nptrend*, Nonparametric trend; *IQR*, interquartile range; *CABG*, coronary artery bypass grafting.

### Inpatient Cardiac Costs for Elective Admissions

**Figure 1:**
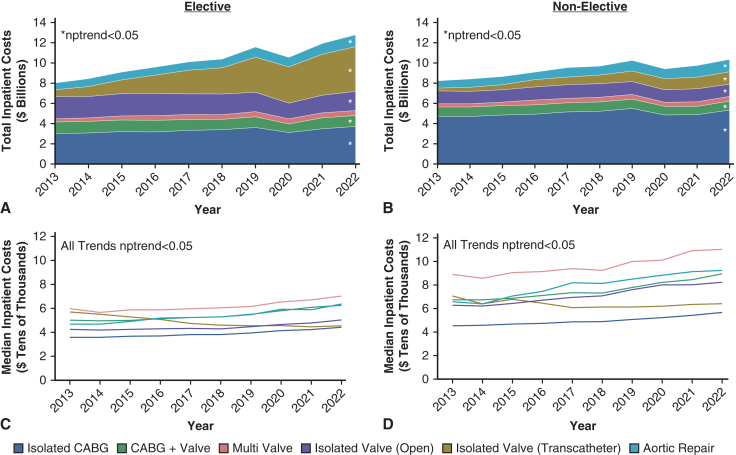
Trends in cumulative and per-admission costs stratified by operation type and admission electivity. *nptrend*, Nonparametric trend; *CABG*, coronary artery bypass grafting.

Annual inpatient costs rose sharply from $8.1 to $12.9 billion over the study period (nptrend < 0.001). Similarly, median per-admission costs increased from $41k [32-56k] to $48k [37-63k] (nptrend < 0.001, Figure 1). Isolated transcatheter valve procedures increased from 6.4% to 38.9% of all cases over the decade (Table 1). The mortality rate among elective operations declined from 2.1% to 1.3% (nptrend < 0.001).

**Figure 2:**
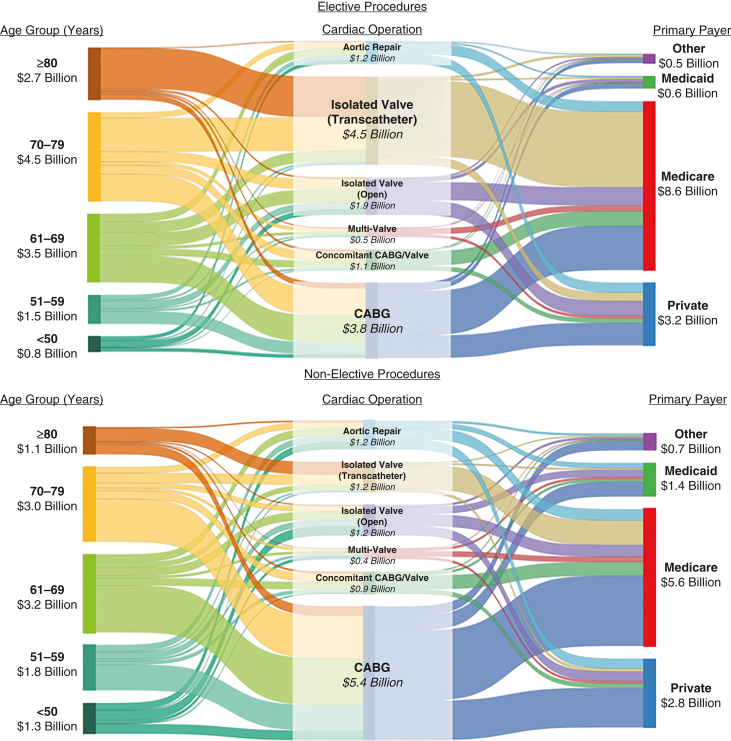
Distribution of 2022 hospitalization costs stratified by elective status. *CABG*, Coronary artery bypass grafting.

In 2022 (n = 233,445), the age group incurring the most costs was 70 to 79 years (35.1%). Isolated transcatheter valve operations were the most common (34.7%) procedure. Lastly, Medicare accounted for the largest proportion of expenditures among all payers at 66.7% (Figure 2).

### Inpatient Cardiac Costs for Nonelective Admissions

Inpatient costs increased from $8.3 billion in 2013 to 10.4 billion in 2022 among nonelective admissions (nptrend = 0.01). Median per-admission costs also increased over the study from $51k [38k-72k] to $63k [$47k-90k] (nptrend < 0.001, Figure 1). Overall mortality rate stayed steady at an annual average of 3.9% (nptrend = 0.78).

Among 2022 admissions (n = 127,910), those aged 61 to 69 years amassed the greatest hospitalization costs (30.8% of total expenditures). CABG comprised the bulk of costs by procedure (51.9%), whereas Medicare accrued the greatest expenditure among all payers (53.8%; Figure 2).

### Factors Associated With Inpatient Costs

**Figure 3:**
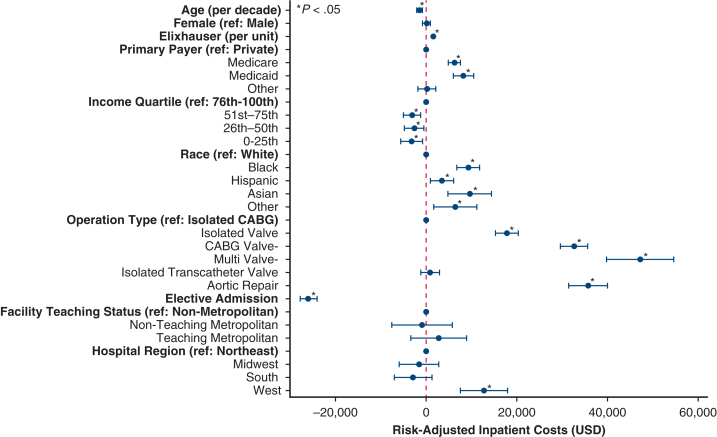
Factors associated with hospitalization costs (2022 alone). *CABG*, Coronary artery bypass grafting; *USD*, US dollars.

After risk-adjustment, greater comorbidity burden (Elixhauser: β $1.5k per unit; 95% CI, 1.2-1.9k), Black race (β $9.3k; 95% CI, 6.7-11.8k; ref: White), and care in the Western United States (β $12.8k; 95% CI, 7.6-17.9k; ref: Northeast) were linked with increased inpatient costs. Conversely, elective admission (β −$26.0k; 95% CI, −27.9, −24.2k), increasing age (β −$1.4k per decade, 95% CI, −2.1, −0.8k), and lowest income quartile (β −$3.2k; 95% CI, −5.7, −0.8k; ref: highest), were linked with a decrement in costs (Figure 3).

## Discussion

With inherently high acuity, cardiac surgery demands significant inpatient resource use, making careful examination of costs a necessity. In this national retrospective analysis, we report a significant increase in inpatient costs for cardiac surgery from 2013 to 2022, even after adjustment for inflation. Although median costs rose for nearly every operation, transcatheter valve procedures not only decreased in per-admission costs but also saw nearly a 6-fold increase in use. We also noted a decline in mortality among elective, but not nonelective procedures, over the last decade. Given their significant implications toward public policy and resource allocation, these findings warrant further discussion.

In the present study, we note a significant increase in hospitalization costs among admissions entailing cardiac surgery. This observation is consistent with several other reports over the previous decade.^3^^,^^11^^,^^12^ Although increasing costs are expected in the face of the aging US population and increasing burden of comorbidities, concerns regarding the sustainability of this growth are warranted.^11^^,^^13^, ^14^, ^15^ Paradoxically, Treffalls and colleagues^16^ found Medicare reimbursements to have declined by >30% over the past 2 decades, amounting to an annual decrement of nearly 2%. This is especially salient given our finding that ∼60% of patients undergoing cardiac surgery are Medicare beneficiaries, which is only expected to increase.^17^^,^^18^ With this divergence in reimbursement rates and costs, disparities in access to quality cardiac surgical care will likely worsen. Although the lowest income quartile was associated with slightly lower adjusted costs, this effect was small and likely reflects collinearity between income, race, and regional factors rather than a meaningful difference in resource use. Taken together, these findings suggest that socioeconomic and racial inequities remain deeply intertwined. Minority-serving and rural hospitals receive disproportionately lower reimbursement for complex cardiac surgical care. As costs continue to increase while reimbursements decline, these centers may face increasing financial strain that jeopardizes equitable access to high-quality surgery. Addressing this imbalance through reimbursement reform specific to high-acuity surgical care will be critical to sustaining the national cardiac surgery infrastructure.^16^^,^^19^^,^^20^ Prompt and strategic budget restructuring, particularly at the federal level, is imperative. Although some have called upon the Society of Thoracic Surgeons to confront this challenge, the issue remains unresolved. In the absence of meaningful reform to the reimbursement of cardiac surgery care, the increasing inpatient costs highlighted in the present study threaten equitable care and the infrastructure of cardiac surgery. These findings underscore the need for evidence-based reform to ensure the sustainability of cardiac surgical care. Aligning reimbursement with resource use and supporting centers that serve high-risk or underserved populations may represent important steps toward equitable and cost-efficient care.

Over the study period, the use of transcatheter valve procedures increased dramatically. The observed change in practice is likely a result of evolving evidence and shifting attitudes toward transcatheter operations, most notably, transcatheter aortic valve replacement (TAVR). Importantly, TAVR was only considered for poor surgical candidates in the early years of the present study, whereas the technology was approved for all risk categories beginning in 2018.^4^^,^^21^ Although transcatheter valve operations differ fundamentally from open surgery, their inclusion was essential to reflect the true evolution of cardiac care in the United States. With maturation of TAVR technology and improving safety profile, an increasing number of patients are now deemed candidates.^4^ In the short term, TAVR has been linked with reduced rates of complications, lower mortality, and shorter length of stay compared with traditional surgical aortic valve replacement.^22^ However, longitudinally, the transcatheter approach may more often require reintervention, potentially incurring greater costs.^22^ Ongoing study and long-term data are needed to provide greater insight into the comparative effectiveness, durability, and cost-efficiency of these procedures.

Congruent with previous reports, we identified a steady decline in rates of mortality among elective operations.^14^^,^^23^ This decline likely reflects improvements in procedural safety and case selection, including the increased adoption of transcatheter valve operations, which have been associated with lower short-term mortality in prior national studies. In the context of increasing costs, adoption of new technologies such as robotic cardiac surgery may have contributed to both improved survival and increased expenditures.^24^^,^^25^ For example, although linked with better short-term outcomes, the cost discrepancy between open and robotic-assisted approaches to mitral valve repair has been reported to be as much as $10,500 per patient.^26^^,^^27^ It remains plausible that advancements in technology and widespread adoption may ultimately result in cost-effective solutions in the long term. Previous work from our group has demonstrated increasing facility volume for robot-assisted mitral valve repair to be inversely associated with costs, suggesting that procedural throughput may need to cross a threshold to achieve cost-effectiveness.^26^ At the national level, this is further supported by the trends in costs of transcatheter procedures observed in the present study, where increased use was accompanied by a decline in per-admission costs. Taken together, these findings highlight the complex interplay between innovation, outcomes, and efficiency and underscore the promising potential to mitigate costs while improving survival in our field.

The present study has several important limitations. First, we are restricted by the limitations of the NIS, which is subject to differential coding practices in addition to the potential for under and erroneous coding. Moreover, there is limited granularity for risk-adjusted models, most notably the NIS lacks laboratory values and imaging findings. The NIS captures only facility-level costs and does not include physician professional fees. Furthermore, because the dataset relies on *International Classification of Diseases* rather than Current Procedural Terminology codes, procedural specificity may be limited. Lastly, the NIS permits regional but not state- or city-level analyses, precluding more granular comparisons across metropolitan areas such as Los Angeles and New York City. Nonetheless, we use both inflation adjustment and multivariable risk-adjustment to make meaningful comparisons of costs in cardiac surgery over the last decade in a national sample.

In a nationally representative cohort of patients undergoing cardiac surgery, we identified an increase in both cumulative and per-admission hospitalization costs. Interestingly, although per-admission spending rose for all other procedures, the median costs of transcatheter valve operations decreased over the study period. Finally, we report a decrement in mortality among those admitted electively. Together, these findings highlight the evolving landscape of cardiac surgery, emphasizing the need for innovation and policy reform to ensure sustainable, high-quality, and equitable care for the future.

### Audio

You can listen to the discussion audio of this article by going to the supplementary material section below.

## Conflict of Interest Statement

The authors reported no conflicts of interest.

The *Journal* policy requires editors and reviewers to disclose conflicts of interest and to decline handling or reviewing manuscripts for which they may have a conflict of interest. The editors and reviewers of this article have no conflicts of interest.

## Audio

Audio 1 This is an audio recording of the presentation of the associated abstract and discussion. The abstract presentation ends at 9:30 and the discussion begins at 9:38.
